# Supplementary material for: The role of a water bug, Sigara striata, in freshwater food webs
Source: PeerJ. 2014 May 22;2:e389. doi: 10.7717/peerj.389 (PMC4034595; doi:10.7717/peerj.389)
Supplement: Supplemental Information [file peerj-02-389-s001.zip › Klecka_Supplementary_Information/Supplementary_File_1.pdf]

The role of a water bug, *Sigara striata*, in freshwater food webs

Jan Klecka

April 28, 2014

Laboratory of Theoretical Ecology, Biology Centre of the Academy of Sciences of the Czech Republic, v.v.i., Institute of Entomology, České Budějovice, Czech Republic

Department of Fish Ecology and Evolution, Eawag: Swiss Federal Institute of Aquatic Science and Technology, Kastanienbaum, Switzerland

Supplementary File 1

Electronic Supplementary Material contains a script to be run in R, files with the specification of models for Bayesian estimation of parameters affecting mortality rates of *Sigara* and its prey and a complete set of raw data. R and JAGS need to be installed, as well as a package rjags for R which allows to control JAGS from R. Files containing specifications of statistical models are provided with the extension .bug; they can be opened and modified by any text editor. The list of files is provided below:

1. Klecka_script.r - a script to perform the analyses reported in the main text. The script is divided in five sections corresponding to individual analyses. Each section can be run separately. Running the script estimates parameter values, plots their posterior distributions, autocorrelation and computes convergence diagnostics of MCMC chains. Note that the script uses 10^5 steps in the MCMC chains to speed up the analysis, if a reader wishes to run the script as is.

2. Sig.mort.full.csv - raw data showing the number of individuals of *Sigara striata* consumed in all replicates of the experiment.

3. Sig.mort.mean.csv - mean mortality rate of *Sigara striata* caused by individual predator species.

4. Sigara_traits.csv - input data for the models of the dependence of *Sigara* mortality on predator traits.

5. Sig.prey.csv - raw data showing mortality of six invertebrates alone and in the presence of 10 individuals of *Sigara striata*.

6. prey.bug - a file specifying the model of the dependence of the mortality of six invertebrates on the presence of *Sigara*.

7. predator_species.bug - model of the mortality of *Sigara* caused by 13 predatory aquatic insects.

8. traits_mass.bug - model of the dependence of the mortality of *Sigara* on body mass of its predators.

9. traits_full.bug - model of the dependence of *Sigara* mortality on all predator traits that were measured. This is the full model reported in the main text.

10. traits_reduced.bug - a simplified version of the previous model excluding a quadratic term of predator body mass and predator microhabitat preference; these parameters had most values close to zero in the full model.
